# Supplementary material for: Mechanism of Huanglian Wendan Decoction in ameliorating non-alcoholic fatty liver disease via modulating gut microbiota-mediated metabolic reprogramming and activating the LKB1/AMPK pathway
Source: PLoS One. 2025 Sep 2;20(9):e0331303. doi: 10.1371/journal.pone.0331303 (PMC12404375; doi:10.1371/journal.pone.0331303)
Supplement: S1 Table — (DOCX) [file pone.0331303.s001.docx]

**1.1 Ingredient identification analysis**

The UPLC-Q-TOF-MS analysis of HLWDD yielded the total ion current chromatogram. In conjunction with databases such as Chemspider and MassBank, a total of 58 chemical components were identified from HLWDD; the specific details are displayed in Table. These included two types of amino acids and their derivatives (1, 3), two types of nucleosides and their derivatives (2, 58), seven types of phenolic acids (5, 6, 8, 9, 11, 18, 23), twenty-five types of flavonoids and their glycosides (12-17, 22, 24, 26, 27, 30, 33, 34, 36-38, 40-44, 49, 53, 54), six types of alkaloids (7, 19, 29, 25, 32, 52), one type of terpenoid (35), eight types of coumarins and furanocoumarins (31, 39, 45, 46, 50, 51, 55, 57), two types of fatty acids and carboxylic acids (4, 28), and five types of others (20, 21, 47, 48, 56). Detailed information on the identified compounds, including retention times, mass spectral data, is provided in Supplementary Materials (Table S1).

**Table S1** Information on chemical ingredients in HLWDD

| **NO** | **Compound** | **Measured**  **value** | **Theoretical**  **value** | **mode** | **Adducts** | **Formula** | **Fragment ion**  **( m/z)** | **References** |
| --- | --- | --- | --- | --- | --- | --- | --- | --- |
| **1** | L-PROLINE | 116.0707 | 116.0706 | pos | M+H | C5H9NO2 | 70.067 | [1] |
| **2** | Cytidine | 112.0507 | 112.0505 | pos | M+H | C9H13N3O5 | 95.0246 | [2] |
| **3** | L-Leucine | 132.1019 | 132.1019 | pos | M+H | C6H13NO2 | 86.0971 | [2] |
| **4** | Succinic acid | 117.0192 | 117.0193 | neg | M-H | C4H6O4 | 73.0292 | [3] |
| **5** | Gallic acid | 169.0149 | 169.0143 | neg | M-H | C7H6O5 | 151.0374 | [4] |
| **6** | Vanillic acid | 167.0349 | 167.0358 | neg | M-H | C8H8O4 | 77.0468 | [5] |
| **7** | Higenamine | 272.1279 | 272.1279 | pos | M+H | C16H17NO3 | 255. 1005, 161. 0539, 107. 0498 | [6] |
| **8** | Cryptochlorogenic acid | 353.0879 | 353.0883 | neg | M-H | C16H18O9 | 191.0554, 135.0440 | [7] |
| **9** | Syringic acid | 197.0457 | 197.0449 | neg | M-H | C9H10O5 | 166 | [8] |
| **10** | Liquiritin | 417.1202 | 417.1176 | neg | M-H | C21H22O9 | 255.0651, 135.0079, 119.0496 | [9] |
| **11** | Caffeic acid | 179.03455 | 180.0425 | neg | M-H | C9H8O4 | 135.0443 | [10] |
| **12** | Rhoifolin | 577.1582 | 577.1562 | neg | M-H | C27H30O14 | 269.045 8 | [11] |
| **13** | Naringenin | 273.0749 | 273.0757 | pos | M+H | C15H12O5 | 153.0160, 119.0490 | [12] |
| **14** | Eriocitrin | 595.1678 | 595.1668 | neg | M-H | C27H32O15 | 151.0029, 135.0445 | [13] |
| **15** | Rutin | 611.1604 | 610.1529 | pos | M+H | C27H30O16 | 303.0491 | [14] |
| **16** | Quercetin | 303.0488 | 303.0498 | pos | M+H | C15H10O7 | 153.0171 | [15] |
| **17** | vitexin | 431.0993 | 431.0997 | neg | M-H | C21H20O10 | 311.055 7, 283.060 9 | [16] |
| **18** | 4-Hydroxybenzoic acid | 137.0243 | 137.0261 | neg | M-H | C7H6O3 | 93.0348 | [5] |
| **19** | Berberrubine | 322.1065 | 322.1 | pos | M+H | C19H15NO4 | 307.05 | [17] |
| **20** | Taxifolin | 303.0515 | 303.0515 | neg | M-H | C15H12O7 | 285.1491 | [18] |
| **21** | Naringin | 579.1703 | 579.1503 | neg | M-H | C27H32O14 | 119.0490 271.0610 | [19] |
| **22** | Diosmin | 607.1689 | 607.1668 | neg | M-H | C28H32O15 | 299.0569, 284.0334 | [20] |
| **23** | Sinapic acid | 223.0614 | 223.0612 | neg | M-H | C11H12O5 | 193.0183 | [21] |
| **24** | Isorhamnetin | 317.0647 | 317.3806 | pos | M+H | C16H12O7 | 302.3412 | [22] |
| **25** | Epiberberine | 336.1223 | 336.1236 | pos | M+ | C20H18NO4+ | 320.0926, 292.0961 | [23] |
| **26** | Hesperidin | 609.1852 | 609.1461 | neg | M-H | C28H34O15 | 151.0037 | [24] |
| **27** | Diosmetin | 301.0704 | 301. 0628 | pos | M+H | C16H12O6 | 284.0311, 256.0361 | [25] |
| **28** | Azelaic acid | 187.0976 | 187.0977 | neg | M-H | C9H16O4 | 125.0969 | [26] |
| **29** | Berberine | 336.1221 | 336.1238 | pos | M+ | C20H18NO4+ | 321.0997, 321.0918, 306.0772 | [27] |
| **30** | Isoliquiritigenin | 257.0806 | 257.081 | pos | M+H | C15H12O4 | 137.0244 | [5] |
| **31** | Xanthotoxol | 203.0339 | 203.0338 | pos | M+H | C11H6O4 | 175.0390, 147.0438, 159.0440 | [11] |
| **32** | Palmatine | 352.1549 | 352.1546 | pos | M+ | C21H22NO4+ | 322.1607, 308.1273 | [28] |
| **33** | Ononin | 431.1335 | 431.1341 | neg | M+H | C22H22O9 | 269.0810, 254.0573 | [29] |
| **34** | Isoliquiritin | 419.133 | 419.144 | neg | M-H | C21H22O9 | 137.0248, 147.0454, 257.0813 | [5] |
| **35** | Obacunone | 455.20597 | 455.2064 | pos | M+H | C26H30O7 | 161.0597 | [5] |
| **36** | Linarin | 593.1861 | 593.1863 | neg | M+H | C28H32O14 | 447.1292, 285.0766, 270.0522 | [30] |
| **37** | Isosakuranetin | 287.091 | 287.0914 | neg | M+H | C16H14O5 | 287.0912, 153.0183 | [9] |
| **38** | Formononetin | 267.0662 | 267.0673 | neg | M-H | C16H12O4 | 252.0428 | [31] |
| **39** | Bergaptol | 203.0337 | 203.0342 | pos | M+H | C11H6O4 | 131.0509, 147.0452 | [32] |
| **40** | calycosin | 285.0742 | 285.0748 | pos | M+H | C16H12O5 | 171.0275 | [33] |
| **41** | Medicarpin | 269.0823 | 269.073 | neg | M-H | C16H14O4 | 269.0438, 254. 0567 | [34] |
| **42** | Hispidulin | 299.0565 | 299.0564 | neg | M-H | C16H12O6 | 284.0317 | [25] |
| **43** | Apigenin | 269.0454 | 269.0457 | pos | M+H | C15H10O5 | 269.0430, 225.0556 | [35] |
| **44** | Irigenin | 359.0751 | 360.32 | neg | M-H | C18H16O8 | 344.0539 | [36] |
| **45** | 7-Hydroxycoumarin | 163.0387 | 163.0858 | pos | M+H | C9H6O3 | 135 | [37] |
| **46** | Bergapten | 217.0494 | 217.0498 | pos | M+H | C12H8O4 | 174.0315 | [38] |
| **47** | Murrayone | 259.0964 | 259.1 | pos | M+H | C15H14O4 | 146.93 | [39] |
| **48** | Eupatilin | 343.0832 | 343.0812 | neg | M-H | C18H16O7 | 328.0567, 298.0162 | [40] |
| **49** | Pectolinarigenin | 313.0723 | 313.0712 | neg | M-H | C17H14O6 | 255.0293, 283.0243 | [41] |
| **50** | Isomeranzin | 261.1116 | 261.1125 | pos | M+H | C15H16O4 | 189.0549, 131.0494 | [40] |
| **51** | 7-Demethylsuberosin | 231.1014 | 231.1016 | pos | M+H | C14H14O3 | 175.0392, 147.0441 | [42] |
| **52** | Coptisine | 320.0918 | 320.0923 | neg | M-H | C19H14NO4 | 318.0737, 308.1268 | [43] |
| **53** | Tangeretin | 373.1279 | 373.1282 | pos | M+H | C20H20O7 | 358.1045, 343.0812 | [44] |
| **54** | Nobiletin | 403.1383 | 403.1389 | pos | M+H | C21H22O8 | 388.1155, 373.0919 | [44] |
| **55** | Osthole | 245.117 | 245.1175 | pos | M+H | C15H1603 | 189.0554, 131.0498 | [45] |
| **56** | BGardenin B | 359.106 | 359.112 | pos | M+H | C19H18O7 | 310.0079 | [46] |
| **57** | Scopoletin | 193.0499 | 193.0498 | pos | M+H | C10H8O4 | 178.0237, 133.0577, 150.0293 | [47] |
| **58** | ADENOSINE | 268.1039 | 268.1031 | pos | M+H | C10H13N5O4 | 136.0616 | [32] |

References

1. Liu, J.X., Li, J.H., Du, C.H., Yan, Y., 2022. Metabonomic study of biochemical changes in serum of PCPA-induced insomnia rats after treatment with Suanzaoren Decoction. China Journal of Chinese Materia Medica 47(6), 1632-1641. <https://doi.org/10.19540/j.cnki.cjcmm.20211204.701.>
2. Chen, Y.Z., Zhou, X.Z., Guo, Y.M., Pan, J.H., Xu, C.Z., Wang, Y., Guo, R., Huang, R.Q., Li, W., Zhao, Y.Q., 2024. Study on extraction process of deer sinew at room temperature, chemical composition analysis, and anti-inflammatory and analgesic activities of extract. Chinese Traditional and Herbal Drugs 55(18), 6186-6195. <https://doi.org/10.7501/j.issn.0253-2670.2024.18.009.>
3. Ren, H., Cui, X.M., Hu, J., Liu, X.M., Chen, Z.Y., 2021. Analysis on chemical constituents in rhizomes of Bergenia scopulosa by UHPLC-Q exactive focus MS/MS. Chinese Journal of Experimental Traditional Medical Formulae 27(09), 118-128.
4. Balkrishna, A., Joshi, M., Tomer, M., Verma, S., Gujral, S., Mulay, V.P., Srivastava, J., Varshney, A., 2022. Identification, validation and standardization of bioactive molecules using UPLC/MS-QToF, UHPLC and HPTLC in Divya-Denguenil-Vati: a Penta-herbal formulation for dengue fever. Chromatographia 85(9), 831-850. <https://doi.org/10.1007/s10337-022-04183-7.>
5. Wang, J., 2023. Study on the material basis of Qianghuo Shengshi Decoction in the treatment of rheumatoid arthritis. Zhejiang University, Hangzhou.
6. Lin, M.T., Zhang, C., Tang, S.L., Zhao, Z.Z., Chen, H.B., Zhang, J.Y., 2015. Metabolites Identification for Alkaloids from Nelumbinis Plumula in Caco-2 Cells by LC/MS/MS. Taditional Chinese Medicine 38(12), 2531-2534.
7. Su, H.N., Li, X.X., Li, Y., Kong, Y.L., Lan, J.L., Huang, Y.F., Liu, Y., 2023. Chemical profiling and rapid discrimination of Blumea riparia and Blumea megacephala by UPLC-Q-Exactive-MS/MS and HPLC. Chin. Herb. Med. 15(2), 317-328. <https://doi.org/10.1016/j.chmed.2022.06.009.>
8. Chen, Y.Y., Cai, X.J., Li, G.W., He, X.Y., Yu, X.X., Yu, X.W., Xiao, Q.M., Xiang, Z., Wang, C.J., 2021. Chemical constituents of radix Actinidia chinensis planch by UPLC-QTOF-MS. Biomed. Chromatogr. 35(7), e5103. <https://doi.org/10.1002/bmc.5103.>
9. Zhang, X., 2018. The study of metabolism of main flavonoids of traditional chinese medicine ingredients In Vitro and In Vivo and qualitative and quantitative analysis of chemical composition for Commelina Communis L.based on LC-MS technology. Hebei Medical University, Shijiazhuang.
10. Wang, H.F., Wang, P., Wang, F., Chen, H.P., Chen, L., Hu, Y., Liu, Y.P., 2024. Integrated HS-GC–IMS and UPLC-Q-Orbitrap HRMS-based metabolomics revealed the characteristics and differential volatile and nonvolatile metabolites of different citrus peels. Curr. Res. Food Sci. 8, 100755. <https://doi.org/10.1016/j.crfs.2024.100755.>
11. Liu, X.M., Yang, Y.Y., Hu, J., Ren, H., Cui, X.M., Tao, H.X., Chen, Z.Y., 2020. Identification of chemical constituents in fengliaoxing fengshi dieda wine. China Pharmacy 31(20), 2473-2480.
12. Liang, Y.T., Wang, J.T., Su, W.W., Xing, L.G., Yao, H.L., Lin, Q.H., Peng, W., 2018. Chemical constituents of Premna fulva Craib by UFLC-Triple TOF-MS/MS. Central South Pharmacy 16(10), 1369-1373.
13. Luo, X., Bao, Y.R., Li, T.J., Wang, S., Han, L., Han, X.N., Meng, X., 2023. Research on quality analysis method of Aurantii Fructus based on “quality-quantity” double standard. Chinese Traditional and Herbal Drugs 54(22), 7293-7299.
14. Zhao, L., Zhong, W.L., Kong, X.R., Kang, Q.Z., Hao, L.M., Zhu, J.Q., Lu, J.K., 2024. Profiling the chemical properties of Foeniculum vulgare Mill. and its flavonoids through comprehensive LC-MS/MS to evaluate their anti-motion sickness effect. Fitoterapia 173, 105816. <https://doi.org/10.1016/j.fitote.2023.105816.>
15. Guo, F.Q., Yang, Y.C., Duan, Y., Li, C., Gao, H.M., Liu, H.Y., Cui, Q.P., Guo, Z.Y., Liu, X.Q., Wang, Z.M., 2023. Quality marker discovery and quality evaluation of Eucommia ulmoides pollen using UPLC-QTOF-MS combined with a DPPH-HPLC antioxidant activity screening method. Molecules 28(13), 5288. <https://doi.org/10.3390/molecules28135288.>
16. Han, Z.L., Shen, L.P., Jiang, Y., Liu, L.S., 2024. Discussion on potential mechanism of Yiqi Yangyin prescription in treating non small cell lung cancer based on UPLC-Q-TOF-MS combined with network pharmacology and molecular dcking techniques. New Chinese Medicine 56(20), 156-168. <https://doi.org/10.13457/j.cnki.jncm.2024.20.030.>
17. Tong, Q.L., Luo, D., Xiang, Z.N., Zhang, Y.L., He, J.X., Hu, Z.F., Xia, R.F., Wu, J.L., Fu, X.N., Li, Q., 2023. Metabolic profiling integrated with pharmacokinetics to reveal the material basis of Xiaokeyinshui extract combination in the treatment of type 2 diabetes in rats. J. Pharm. Biomed. Anal. 225, 115224. <https://doi.org/10.1016/j.jpba.2022.115224.>
18. Moura, F.C.S., dos Santos Machado, C.L., Paula, F.R., Couto, A.G., Ricci, M., Cechinel-Filho, V., Bonomini, T.J., Sandjo, L.P., Bresolin, T.M.B., 2021. Taxifolin stability: In silico prediction and In vitro degradation with HPLC-UV/UPLC–ESI-MS monitoring. J. Pharm. Anal. 11(2), 232-240. <https://doi.org/10.1016/j.jpha.2020.06.008.>
19. Jackson Seukep, A., Zhang, Y.L., Xu, Y.B., Guo, M.Q., 2020. In vitro antibacterial and antiproliferative potential of Echinops lanceolatus Mattf.(Asteraceae) and identification of potential bioactive compounds. Pharmaceuticals 13(4), 59. <https://doi.org/10.3390/ph13040059.>
20. Zhang, X.M., 2018. Total flavonoids from Ziziphora clinopodioides Lam pre-vents atherosclerosis by protecting endothelial cells and inhibiting inflammatory reaction and the underlying mechanisms. Xinjiang Medical University, Wulumuqi.
21. Liu, H.H., Li, Z.L., Xia, X.Y., Zhang, R.Y., Wang, W., Xiang, X., 2023. Chemical profile of phenolic extracts from rapeseed meal and inhibitory effects on α-glucosidase: UPLC-MS/MS analysis, multispectral approaches, molecular simulation and ADMET analysis. Food Res. Int. 174, 113517. [https://doi.org/10.1016/j.foodres.2023.113517](https://doi.org/10.1016/j.foodres.2023.113517).
22. Cruz, J.E.R.d., Saldanha, H.C., Nascimento, A.M.d., Borges, R.B., Gomes, M.d.S., Freitas, G.R.O.e., Leal, C.M., Ferreira, E.A., da Silva Filho, A.A., Morais, E.R., 2023. Evaluation of the antioxidant, antimicrobial, and anti-biofilm effects of the stem bark, leaf, and seed extracts from Hymenaea courbaril and characterization by UPLC-ESI-QTOF-MS/MS analysis. Antibiotics 12(11), 1601. [https://doi.org/10.3390/antibiotics12111601](https://doi.org/10.3390/antibiotics12111601).
23. Wang, X.H., 2023. Study on the compatibility effect and material basis of Coptidis Rhizoma - Euodiae Fructus herb pair against sepsis. Shaanxi University of Chinese Medicine, Xian.
24. Li, G., Lei, J., Li, S., Jiang, Y., Zhang, F., Song, C., Xiao, S., Fu, S., Zhou, J., Wu, F., Wang, G., 2022. Extraction of flavonoids from citri reticulatae pericarpium viride using a deep eutectic solvent. RSC Adv. 12(41), 26975-26988. [https://doi.org/10.1039/d2ra04276b](https://doi.org/10.1039/d2ra04276b).
25. Yang, Y.T., Zhang, Q.S., Fu, Y.L., Sun, J., 2021. Components, stability and antioxidant activity of pigment from Dalbergia bariensis heartwood. Scientia Silave Sinicae 57(03), 126-134.
26. Ba, D.L.H., Wu, J.S., Bao, B., Liu, X.W., Ao, D.F., 2024. Analysis of chemical constituents and components absorbed into blood of baidoukou aqueous extract based on UPLC-Q-Exactive-Orbitrap MS/MS. Chinese Journal of Modern Applied Pharmacy, 1-9. [https://doi.org/10.13748/j.cnki.issn1007-7693.20233045](https://doi.org/10.13748/j.cnki.issn1007-7693.20233045).
27. Wang, P., Chen, Y., Peng, J.L., Peng, Q.X., 2024. Anti-breast cancer pharmacodynamic substances and mechanism of action of Alocasia cucullata based on UPLC-Q-TOF-MS/MS and network pharmacology. Journal of Hunan University of Chinese Medicine 44(01), 77-85.
28. Yang, Y., Hao, W.J., Yang, Y.L., Zhang, S.J., Wang, H., Wang, M.X., Dong, T., Shang, Z.P., Yang, W.M., 2022. Chemical profiling and quantification of potential bioactive components in gandouling pill by ultra-high performance liquid chromatography coupled with diode array detector/quadruple-qrbitrap mass spectrometry. Molecules 27(23), 8247. [https://doi.org/10.3390/molecules27238247](https://doi.org/10.3390/molecules27238247).
29. Cui, J.J., Zheng, X.W., Yang, D.L., Hu, Y.H., An, C.M., Bo, Y.F., Li, H.Z., Zhang, Y.L., Niu, M., Xue, X.T., 2019. Astragali radix total flavonoid synergizes cisplatin to inhibit proliferation and enhances the chemosensitivity of laryngeal squamous cell carcinoma. RSC Adv. 9(42), 24471-24482. [https://doi.org/10.1039/c9ra04701h](https://doi.org/10.1039/c9ra04701h).
30. Li, L., Zhang, C., Zheng, W., Yu, Y., Zhang, F., Gao, H., 2021. UPLC-QqQ-MS-based quantitative analysis on changes of ten constituents between the raw and salt-water processed Phellodendron chinense. Chinese Traditional Patent Medicine 43(11), 3082-3088.
31. Luo, X.Q., 2019. Studies on components and pharmacodynamics of fresh-cuting pieces and traditional pieces of Aurantii Fructus. Jiangxi University of Traditional Chinese Medicine, Nanchang.
32. Li, X.J., Jiang, Z.Y., Xiao, Z., Chen, X.F., Wang, S.M., Zhang, Y.X., Pu, W.Y., 2024. Identification of chemical components of Longmu Qingxin Mixture by UPLC-Q-TOF-MS and research on its material basis for attention deficit hyperactivity disorder. Chinese Traditional Patent Medicine 46(02), 490-498. https://doi.org/10.3969/j.issn.1001-1528.2024.02.023.
33. Wu, J.Y., Cai, B.R., Zhang, A., Zhao, P., Du, Y., Liu, X.F., Zhao, D., Yang, L., Liu, X.G., Li, J.S., 2022. Chemical identification and antioxidant screening of Bufei Yishen formula using an offline DPPH ultrahigh-performance liquid Chromatography Q-extractive orbitrap MS/MS. Int. J. Anal. Chem. 202
34. Wu, J., Cai, B., Zhang, A., Zhao, P., Du, Y., Liu, X., Zhao, D., Yang, L., Liu, X., Li, J., 2022. Chemical identification and antioxidant screening of Bufei Yishen formula using an offline DPPH ultrahigh-performance liquid chromatography Q-extractive orbitrap MS/MS. Int J Anal Chem 2022, 1423801. <https://doi.org/10.1155/2022/1423801.>
35. Cui, L.J., Ma, C., Shi, W.Q., Yang, C., Wu, J.P., Wu, Z.H., Lou, Y.F., Fan, G.R., 2023. A systematic study of Yiqi Qubai standard decoction for treating vitiligo based on UPLC-Q-TOF/MS combined with chemometrics, molecular docking, and cellular and zebrafish assays. Pharmaceuticals 16(12), 1716. [https://doi.org/10.3390/ph16121716](https://doi.org/10.3390/ph16121716).
36. Mykhailenko, O., Korinek, M., Ivanauskas, L., Bezruk, I., Myhal, A., Petrikaitė, V., El-Shazly, M., Lin, G.-H., Lin, C.-Y., Yen, C.-H., 2020. Qualitative and quantitative analysis of Ukrainian Iris species: A fresh look on their antioxidant content and biological activities. Molecules 25(19), 4588. [https://doi.org/10.3390/molecules25194588](https://doi.org/10.3390/molecules25194588).
37. Liu, Y., Li, H., Chen, L., Zhao, H., Liu, J., Gong, S., Ma, D., Chen, C., Zeng, S., Long, H., 2023. Mechanism and Pharmacodynamic Substance Basis of Raw and Wine‐Processed Evodia rutaecarpa on Smooth Muscle Cells of Dysmenorrhea Mice. Pain Research and Management 2023(1), 7711988.
38. Liu, Y.Q., Li, H., Chen, L., Zhao, H.X., Liu, J., Gong, S., Ma, D.F., Chen, C.M., Zeng, S.Q., Long, H.P., 2023. Mechanism and pharmacodynamic substance basis of raw and wine-processed evodia rutaecarpa on smooth muscle cells of dysmenorrhea mice. Pain Res. Manage. 2023, 7711988. [https://doi.org/10.1155/2023/7711988](https://doi.org/10.1155/2023/7711988).
39. Zhai, S.Y., Deng, X.X., Zhang, C., Zhou, Y., Xie, H.Z., Jiang, Z., Jia, L., 2020. A novel UPLC/MS/MS method for rapid determination of murrayone in rat plasma and its pharmacokinetics. J. Pharm. Biomed. Anal. 180, 113046. [https://doi.org/10.1016/j.jpba.2019.113046](https://doi.org/10.1016/j.jpba.2019.113046).
40. Chen, L., Zhu, Y.Y., Kang, L.P., Guo, C.W., Wang, Y.Q., Li, S.G., HZ, D., Liu, D.H., 2023. Anti-inflammatory material basis and mechanism of Artemisia stolonifera based on UPLC-Q-TOF-MS combined with network pharmacology and molecular docking. China Journal of Chinese Materia Medica 48(14), 3701-3714.
41. Li, C.H., Li, X.C., Zeng, J.Y., Cai, R.X., Chen, S.M., Chen, B., Zhao, X.J., 2024. Detection of adulterated Naodesheng tablet (Naodesheng Pian) via in-depth chemical analysis and subsequent reconstruction of its pharmacopoeia Q-markers. Molecules 29(6), 1392. [https://doi.org/10.3390/molecules29061392](https://doi.org/10.3390/molecules29061392).
42. Hu, A.Y., 2021. Extraction, purification, antioxidation and product prediction of flavonoids from pomelo peel. South China Agricultural University, Guangzhou.
43. Zhang, Y., Deng, Q., Wei, M., Fu, C.Q., Zhang, X., 2022. Rapid identification chemical constituents in Angelica Keiskei based on UPLC-0 orbitrap HRMS technology. Asia-Pacific Traditional Medicine 18(02), 40-49.
44. Li, J., Zou, S., Yang, W., Peng, M., Chen, B., Deng, J., Wei, M., Zheng, G., 2023. Identification of volatile and nonvolatile compounds in Citri Reticulatae Pericarpium Viride using GC–MS, UPLC‐Q‐Exactive Orbitrap‐MS, and HPLC‐PDA. Food science & nutrition 11(3), 1415-1425.
45. Li, J.X., Zou, S.Q., Yang, W.L., Peng, M.D., Chen, B.Z., Deng, J.J., Wei, M.Y., Zheng, G.D., 2023. Identification of volatile and nonvolatile compounds in Citri Reticulatae Pericarpium Viride using GC-MS, UPLC-Q-Exactive Orbitrap-MS, and HPLC-PDA. Food Sci. Nutr. 11(3), 1415-1425. [https://doi.org/10.1002/fsn3.3181](https://doi.org/10.1002/fsn3.3181).
46. Liao, M., 2018. Investigation on therapeutic basis matter of trollius ledebourii and metabolites identificaion of osthole using ultra-high-performance liquid chromatography coupled to quadrupole time-of-flight mass spectrometry. Hebei Medical University, Shijiazhuang.
47. Elgabry, R.M., Hassan, M., Fawzy, G.A., Meselhy, K.M., Mohamed, O.G., Al-Taweel, A.M., Sedeek, M.S., 2024. A comparative analysis of polysaccharides and ethanolic extracts from two Egyptian sweet potato cultivars, Abees and A 195: chemical characterization and immunostimulant activities. Metabolites 14(4), 222. [https://doi.org/10.3390/metabo14040222](https://doi.org/10.3390/metabo14040222).
