# Supplementary material for: Mechanism of Huanglian Wendan Decoction in ameliorating non-alcoholic fatty liver disease via modulating gut microbiota-mediated metabolic reprogramming and activating the LKB1/AMPK pathway
Source: PLoS One. 2025 Sep 2;20(9):e0331303. doi: 10.1371/journal.pone.0331303 (PMC12404375; doi:10.1371/journal.pone.0331303)
Supplement: S2 Table — Full list of 229 targets identified through network pharmacology. (DOCX) [file pone.0331303.s002.docx]

**2.1 Network Pharmacology Analysis**

List of 229 shared targets identified by network pharmacology between HLWDD active compounds and NAFLD-related genes. These targets were obtained through intersecting predicted targets of HLWDD ingredients (via SwissTargetPrediction, SEA, etc.) with NAFLD-related genes collected from databases such as GeneCards and DisGeNET.

**Table S2** Shared targets between HLWDD active compounds and NAFLD identified through network pharmacology analysis.

| No. | Target | No. | Target | No. | Target | No. | Target |
| --- | --- | --- | --- | --- | --- | --- | --- |
| 1 | GABRA2 | 61 | NFE2L2 | 121 | CHEK2 | 181 | CHRM3 |
| 2 | ACE | 62 | STAT3 | 122 | TGM2 | 182 | LIPE |
| 3 | F2 | 63 | TNF | 123 | SIRT3 | 183 | FASN |
| 4 | DPP4 | 64 | XDH | 124 | SIRT1 | 184 | ALPL |
| 5 | ADK | 65 | ALDH2 | 125 | FLT1 | 185 | GCGR |
| 6 | GAPDH | 66 | NOX4 | 126 | ROCK1 | 186 | PON1 |
| 7 | HSPA8 | 67 | TP53 | 127 | SELE | 187 | MAP3K8 |
| 8 | HSPA5 | 68 | CD38 | 128 | AGPAT2 | 188 | BRAF |
| 9 | MAPK1 | 69 | RPS6KA3 | 129 | GSK3A | 189 | PLAU |
| 10 | CA2 | 70 | PLA2G1B | 130 | EPHA2 | 190 | CASP3 |
| 11 | GSK3B | 71 | CES1 | 131 | CCNC | 191 | CASP7 |
| 12 | PTPN1 | 72 | PPARG | 132 | CDK8 | 192 | CYP17A1 |
| 13 | CACNA2D1 | 73 | CES2 | 133 | CFTR | 193 | PRKDC |
| 14 | GRM5 | 74 | RXRA | 134 | FABP4 | 194 | PDE4A |
| 15 | TTR | 75 | MET | 135 | FABP3 | 195 | GSTP1 |
| 16 | IGF1R | 76 | CTSB | 136 | FABP5 | 196 | DGAT1 |
| 17 | SERPINE1 | 77 | INSR | 137 | PPARD | 197 | FAP |
| 18 | KDM6B | 78 | IGFBP3 | 138 | FABP2 | 198 | MAPK9 |
| 19 | FTO | 79 | YWHAG | 139 | PPARA | 199 | JUN |
| 20 | MMP9 | 80 | SIRT2 | 140 | AR | 200 | CXCR3 |
| 21 | MMP1 | 81 | VCP | 141 | VDR | 201 | HMOX1 |
| 22 | MMP2 | 82 | VEGFA | 142 | NR1H4 | 202 | BMP4 |
| 23 | DRD2 | 83 | BCL2 | 143 | GPBAR1 | 203 | HSP90B1 |
| 24 | DRD4 | 84 | CDK4 | 144 | SERPINA6 | 204 | SHH |
| 25 | ADRB2 | 85 | ALOX12 | 145 | NPC1L1 | 205 | CHUK |
| 26 | ADRB1 | 86 | HNF4A | 146 | GSTK1 | 206 | NOS1 |
| 27 | DRD3 | 87 | HRAS | 147 | FDPS | 207 | VCAM1 |
| 28 | DRD1 | 88 | PTGS2 | 148 | FDFT1 | 208 | ALB |
| 29 | SLC6A3 | 89 | GLO1 | 149 | SLC1A3 | 209 | TGFBR1 |
| 30 | ADRB3 | 90 | MPO | 150 | MAPK14 | 210 | IDH1 |
| 31 | SLC6A4 | 91 | CDK1 | 151 | LRRK2 | 211 | CETP |
| 32 | OPRM1 | 92 | AKT1 | 152 | AOC3 | 212 | PHLPP2 |
| 33 | OPRD1 | 93 | AXL | 153 | GRK2 | 213 | NR1H3 |
| 34 | F3 | 94 | AKR1A1 | 154 | CNR2 | 214 | HDAC9 |
| 35 | ESR1 | 95 | PIK3CG | 155 | NOS2 | 215 | KIF11 |
| 36 | MAOA | 96 | APEX1 | 156 | KCNMA1 | 216 | NOS3 |
| 37 | AKR1B10 | 97 | ARG1 | 157 | MGAM | 217 | PRKAB1 |
| 38 | MMP13 | 98 | AHR | 158 | NFKB1 | 218 | BMPR1A |
| 39 | APP | 99 | PARP1 | 159 | CCND1 | 219 | PABPC1 |
| 40 | ELANE | 100 | HDAC8 | 160 | PDGFRB | 220 | RGS4 |
| 41 | PRKCD | 101 | CDC42 | 161 | HSPA1A | 221 | PRMT3 |
| 42 | PRKCA | 102 | RAF1 | 162 | GSR | 222 | NAT1 |
| 43 | ERN1 | 103 | XBP1 | 163 | NQO1 | 223 | THRB |
| 44 | SLC5A2 | 104 | HSP90AA1 | 164 | MTOR | 224 | BRD2 |
| 45 | EIF4A1 | 105 | HSP90AB1 | 165 | MDM2 | 225 | TSPO |
| 46 | EPHX2 | 106 | RELA | 166 | GCK | 226 | FHIT |
| 47 | CYP1B1 | 107 | ICAM1 | 167 | CFD | 227 | DNMT1 |
| 48 | HSD17B1 | 108 | DGAT2 | 168 | FPR2 | 228 | EZH2 |
| 49 | SHBG | 109 | CYP1A1 | 169 | NAMPT | 229 | PRMT7 |
| 50 | MMP7 | 110 | ACLY | 170 | PFKFB3 |  |  |
| 51 | TERT | 111 | CTNNB1 | 171 | CTSS |  |  |
| 52 | HCAR2 | 112 | MAPK8 | 172 | P2RX7 |  |  |
| 53 | MIF | 113 | SLC13A5 | 173 | HTR2A |  |  |
| 54 | TLR4 | 114 | CAMKK2 | 174 | HMGCR |  |  |
| 55 | EGFR | 115 | PRKCE | 175 | CCR1 |  |  |
| 56 | PIK3CB | 116 | TLR9 | 176 | EPHX1 |  |  |
| 57 | CYP1A2 | 117 | PIN1 | 177 | CNR1 |  |  |
| 58 | CYP3A4 | 118 | RPS6KB1 | 178 | HDAC1 |  |  |
| 59 | PIK3CA | 119 | SCD | 179 | NR1I2 |  |  |
| 60 | HSD11B1 | 120 | PIK3CD | 180 | PSMB8 |  |  |
