# Supplementary material for: Mechanism of Huanglian Wendan Decoction in ameliorating non-alcoholic fatty liver disease via modulating gut microbiota-mediated metabolic reprogramming and activating the LKB1/AMPK pathway
Source: PLoS One. 2025 Sep 2;20(9):e0331303. doi: 10.1371/journal.pone.0331303 (PMC12404375; doi:10.1371/journal.pone.0331303)
Supplement: S3 Table — Linear regression equations, precision, repeatability, stability, and recovery results. (DOCX) [file pone.0331303.s003.docx]

**3.1 Methodological Investigation**

Investigation of Linear Relationship: The regression equations, linear ranges, and correlation coefficients for each reference substance were calculated, and the results are shown in Table S2, indicating that the linear relationship of the seven components is good within a certain concentration range.

**Table S3** Linear Regression Data of Seven Components

| Compound | Regression equation | *r*^2^ | Linear range（µg/mL） |
| --- | --- | --- | --- |
| Quercetin | Y = 14647X-55556 | 0.9993 | 11.00-110.0 |
| Epiberberine | Y= 2173.4X-12612 | 0.9995 | 18.00-180.0 |
| Coptisine | Y = 37797X-75837 | 0.9994 | 18.00-180.0 |
| Palmatine | Y= 47808X-90789 | 0.9993 | 13.00-130.0 |
| Berberine | Y = 2046.2X-8890.9 | 0.9994 | 13.00-130.0 |
| Naringenin | Y = 17492X-47532 | 0.9996 | 18.00-180.0 |
| Obacunone | Y= 36168X-66872 | 0.9994 | 15.00-150.0 |

Precision Test: The precision was calculated based on the peak areas of each component. The RSD values for the contents of quercetin, epiberberine, coptisine, palmatine, berberine, naringenin, and obacunone were 0.44%, 0.61%, 0.44%, 0.62%, 1.20%, 1.25%, and 1.05%, respectively, indicating good instrument precision.

Repeatability Test: The contents and their RSD values of each component in six repeated injections of the sample were calculated. The RSD values for quercetin, epiberberine, coptisine, palmatine, berberine, naringenin, and obacunone were 0.49%, 0.51%, 0.38%, 1.46%, 1.56%, 2.17%, and 2.20%, respectively, suggesting good repeatability of the method.

Stability Test: The contents and their RSD values of each component in the sample at 0, 2, 4, 8, 12, and 24 hours were calculated. The RSD values for quercetin, epiberberine, coptisine, palmatine, berberine, naringenin, and obacunone were 0.36%, 0.46%, 0.46%, 0.61%, 0.41%, 0.40%, and 1.62%, respectively, indicating good stability of the sample within 24 hours.

Recovery Test: The contents and recovery rates of each compound in the sample solution after adding corresponding amounts of reference substances were calculated. The average recovery rates for quercetin, epiberberine, coptisine, palmatine, berberine, naringenin, and obacunone were 100.36%, 99.44%, 101.33%, 99.91%, 99.71%, 99.35%, and 99.78%, respectively, with RSD values of 0.65%, 0.67%, 0.71%, 0.59%, 1.53%, 1.40%, and 1.47%, respectively, indicating good accuracy of the method.
