# Supplementary material for: Mechanism of Huanglian Wendan Decoction in ameliorating non-alcoholic fatty liver disease via modulating gut microbiota-mediated metabolic reprogramming and activating the LKB1/AMPK pathway
Source: PLoS One. 2025 Sep 2;20(9):e0331303. doi: 10.1371/journal.pone.0331303 (PMC12404375; doi:10.1371/journal.pone.0331303)
Supplement: Attachment — Submitted filename: Reviewer comments_Plos.pdf [file pone.0331303.s004.pdf]

Reviewer Comments for Manuscript Number PONE-D-25-23758 "Mechanism of Huanglian Wendan Decoction in Ameliorating Non-Alcoholic Fatty Liver Disease via Modulating Gut Microbiota-Mediated Metabolic Reprogramming and Activating the LKB1/AMPK Pathway"

1. Abstract

Strengths : Clearly presents important topic, comprehensive methodology, mechanistic insight and strong outcome summary

Suggestions for improvement :

The abstract would benefit from clearer segmentation into logical components : Background, objective, methods, key results, conclusion. Research objective need explicit statement. Too many abbreviations may overwhelm readers unfamiliar with them. The final sentence effectively summarize the results, but could emphasize what gap this study fills or how it advances current knowledge.

2. Introduction

Strengths : The introduction provides a well-structured and scientifically grounded rationale for the proposed study. It integrates current knowledge on NAFLD pathogenesis with the potential of Traditional Chinese Medicine (TCM) interventions, specifically Huanglian Wendan Decoction (HLWDD). The writing is rich in mechanistic detail, and the research hypothesis is clearly stated at the end. However, there are areas that could be improved for clarity, focus, and better alignment with the journal criteria.

Suggestion for improvement :

While a hypothesis is stated, a specific research question is not directly framed in a question format. It is recommended to clearly formulate the research aim or question (e.g., “Does HLWDD ameliorate NAFLD through modulation of the gut-liver axis and activation of the LKB1/AMPK pathway?”).

The introduction is overly dense, covering too many compounds and mechanisms, which may dilute the main focus on HLWDD. Suggested improvement: Briefly summarize the rationale for prior TCM components, then clearly narrow down the novelty and justification for choosing HLWDD.

While the text implies the gap (limited translation of probiotics, complexity of AMPK activators, etc.), it does not explicitly state what specific knowledge gap the current study addresses. A stronger opening or closing paragraph could highlight: “Despite evidence for the metabolic and microbiota-modulating effects of HLWDD constituents, the precise mechanisms through which HLWDD influences the gut-liver axis and AMPK signaling in NAFLD remain unclear.”

The contribution to scientific knowledge is implied but not explicitly declared. Clarify what novel insight this study will offer (e.g., “This study will elucidate the synergistic effects of HLWDD on microbial metabolites and hepatic energy metabolism, providing a novel therapeutic avenue for NAFLD.”)

3. Methods

Strengths : This Materials and Methods section reflects a well-designed and multifaceted experimental strategy that integrates modern systems biology with traditional medicine research.

Suggestion for improvement :

There are multiple grammatical inconsistencies, awkward phrasing, and redundancies throughout. Example: “...use used to screen...” → should be “used to screen”. Suggestion : Comprehensive language editing is needed to meet international publication standards.

The section is overly lengthy and lacks clear subsections, making it difficult to navigate.

Suggestion: Divide into well-labeled subsections such as:

Reagents and Chemicals

Preparation of HLWDD

Quantification of Core Components

Network Pharmacology and Molecular Docking

Animal Experimentation

Histological and Biochemical Analysis

Microbiome and Metabolomic Analysis

Statistical Analysis

Primer sequences are referred to in Table 3, but the actual table is not included.

Positive drug details (e.g., purity and brand of metformin) are not mentioned.

The exact number of rats used per group post-modeling isn't clearly stated after accounting for attrition.

Quality control parameters for the LC-MS/MS (e.g., resolution, scan range, ionization mode) are not fully described.

Preparation steps for HLWDD decoction and ethanol precipitation are wordy and at times unclear (e.g., “accurately measure 2 mL... take the filtrate and you have it”).

Suggestion: Use stepwise or bulleted protocol format and include specific parameters (e.g., temperature of refrigeration, type of ethanol).

While software used is listed, the rationale for choosing certain tests (e.g., LSD-t test instead of Tukey) is not explained.

Suggestion: Justify the choice of tests and confirm assumption checks (e.g., normality, homogeneity of variance).

Some materials (e.g., primer sources) are repeated unnecessarily.

Suggestion: Consolidate repeated supplier information or group by function.

Result :

Strengths : This Results section is comprehensive, well-organized, and logically structured, covering multiple layers of evidence (chemical profiling, network pharmacology, molecular docking,

quantitative validation, in vivo assays, and multi-omics analysis). It successfully integrates phytochemical analysis with biological and pharmacological relevance to NAFLD, which enhances the translational impact of the work.

Suggestion for improvement :

Clarify if identification was based on MS/MS fragmentation or just database matching

Example : "A total of 58 compounds were identified based on retention times and MS/MS spectra, as referenced in ChemSpider and MassBank databases (Table S1; Supplementary Table S1)."

Consider adding a supplementary table listing all 229 shared targets for transparency.

Consider stating whether these seven compounds were chosen based on pharmacological relevance or abundance.

Metformin is mentioned but not introduced earlier—clarify its role as a positive control

Clearly separate qPCR vs. WB results (e.g., "Protein levels assessed by WB showed...").

Use more precise statistical notation; avoid duplication (e.g., "was significantly downregulated (P < 0.05), and significantly regulated...").

Discussion :

Strengths : The discussion is well-structured, provides mechanistic insight, and draws appropriate connections between experimental findings and current literature.

Suggestions for improvement :

The text frequently suggests causal relationships (e.g., "HLWDD controls intestinal flora metabolic reprogramming") based on associative findings in an animal model.

Suggestion: Use more cautious language such as "may regulate," "is associated with," or "suggests potential modulation" unless causality is experimentally confirmed.

The discussion lacks acknowledgment of key limitations, such as:

Use of a single animal model

Absence of dose-response data or comparison with known therapies

Lack of functional validation (e.g., using inhibitors of AMPK or germ-free animals)

Suggestion: Add a paragraph critically acknowledging these limitations and propose future directions.

The paragraph on aspartate metabolism and its bidirectional relationship with LKB1/AMPK is dense and hard to follow. Suggestion: Break down complex mechanisms into simpler sentences.
