# Supplementary material for: Mechanism of Huanglian Wendan Decoction in ameliorating non-alcoholic fatty liver disease via modulating gut microbiota-mediated metabolic reprogramming and activating the LKB1/AMPK pathway
Source: PLoS One. 2025 Sep 2;20(9):e0331303. doi: 10.1371/journal.pone.0331303 (PMC12404375; doi:10.1371/journal.pone.0331303)
Supplement: Attachment — Submitted filename: Response to Reviewers for Manuscript PONE.docx [file pone.0331303.s006.docx]

Response to Reviewers for Manuscript PONE-D-25-23758

Title: Mechanism of Huanglian Wendan Decoction in Ameliorating Non-Alcoholic Fatty Liver Disease via Modulating Gut Microbiota-Mediated Metabolic Reprogramming and Activating the LKB1/AMPK Pathway

We sincerely thank the reviewers for their thorough and constructive feedback. We have carefully addressed all comments and revised the manuscript accordingly. Below is a detailed point-by-point response.

Reviewer Comments and Author Responses

1. Abstract

Comment: Abstract lacks clear segmentation; too many abbreviations; objective not explicit; final sentence could emphasize novelty.

Response: Thank you for this suggestion. We have revised the abstract to clearly segment it into Background, Objective, Methods, Key Results, and Conclusion. We reduced the number of abbreviations and explicitly stated the research objective: "This study aims to investigate whether HLWDD can alleviate NAFLD by modulating gut microbiota-mediated metabolism and activating the LKB1/AMPK pathway." The final sentence now emphasizes the novel contribution of elucidating a gut microbiota–metabolite–signaling axis in HLWDD’s mechanism.

2. Introduction

Comment: A specific research question should be clearly framed. The introduction is too dense and includes too many compounds. The knowledge gap is implied but not clearly stated.

Response: We agree and have revised the introduction accordingly. A specific research question has been added: "Does HLWDD ameliorate NAFLD through modulation of the gut-liver axis and activation of the LKB1/AMPK pathway?" We streamlined the discussion of prior TCM components and narrowed the focus to HLWDD’s novelty. A concise statement of the knowledge gap was added at the end of the introduction, along with a declaration of the study’s expected contribution to scientific understanding.

3. Methods

Comment: Grammar and phrasing need improvement. Subsections should be clearly labeled. Missing details on primers, metformin, LC-MS/MS parameters, and group sizes. HLWDD preparation is unclear. Statistical tests lack justification.

Response:

We performed a comprehensive language edit throughout the Methods section.

Subsections were added: Reagents and Chemicals, Preparation of HLWDD, Quantification of Core Components, Network Pharmacology and Molecular Docking, Animal Experimentation, Histological and Biochemical Analysis, Microbiome and Metabolomic Analysis, and Statistical Analysis.

Primer sequences are now provided in Supplementary Table 3.

Metformin is now described in detail (purity, manufacturer, and dose) as a positive control.

The number of rats per group after attrition is now clearly specified.

Detailed LC-MS/MS parameters (ion mode, scan range, resolution) have been added.

HLWDD preparation steps have been rewritten in stepwise format with specific details.

We now justify statistical test choices and note assumption checks (e.g., for ANOVA, Levene's test for homogeneity).

4. Results

Comment: Clarify whether MS/MS fragmentation or database matching was used. Add a supplementary table listing 229 targets. Justify selection of seven compounds. Clarify metformin role. Separate qPCR and WB results. Improve statistical wording.

Response:

We clarified in the revised manuscript that compound identification was based on both retention time and MS/MS fragmentation spectra, cross-referenced with ChemSpider and MassBank.

A new Supplementary Table S2 has been added to list the 229 shared targets for transparency.

The rationale for selecting the seven compounds is now provided (based on both abundance and pharmacological relevance).

Metformin’s role as a clinically validated positive control is now clearly introduced in the Methods and Results.

WB and qPCR results are clearly separated using structured paragraphs with appropriate headings.

Statistical phrasing has been revised throughout to eliminate redundancy and improve clarity (e.g., "expression was significantly reduced, P < 0.05").

5. Discussion

Comment: Avoid overstating causality from associative data. Acknowledge study limitations. Clarify dense paragraph on aspartate metabolism.

Response:

We have revised all statements implying causality to use cautious language, such as "may regulate," "is associated with," and "suggests."

A new paragraph has been added to explicitly acknowledge study limitations, including use of a single animal model, lack of dose-response assessment, absence of comparison to standard therapies, and need for functional validation using AMPK inhibitors or germ-free models.

The paragraph discussing the bidirectional regulation between aspartate metabolism and LKB1/AMPK has been rewritten into simpler, clearer sentences to aid comprehension.

We appreciate the reviewers’ constructive suggestions and believe that these revisions have substantially improved the quality and clarity of our manuscript. We look forward to your favorable consideration for publication in PLOS ONE.

6.

Acknowledgments

This work was supported by Hunan Provincial Department of Education Research Project (23B0375); Hunan Provincial Administration of Traditional Chinese Medicine Research Project (C2023021); Hunan University of Traditional Chinese Medicine University-level Research Project (2024XJZC017). The funders had no role in study design, data collection and analysis, decision to publish, or preparation of the manuscript.

Institutional Review Board Statement

The study was conducted according to the guidelines of the Declaration of Helsinki, and approved by the Animal Ethics Committee of the Hunan University of Chinese Medicine (LLBH-LL2022111704).

Sincerely,

Jianping Zhu

Corresponding Author
