# Supplementary material for: Mechanism of Huanglian Wendan Decoction in ameliorating non-alcoholic fatty liver disease via modulating gut microbiota-mediated metabolic reprogramming and activating the LKB1/AMPK pathway
Source: PLoS One. 2025 Sep 2;20(9):e0331303. doi: 10.1371/journal.pone.0331303 (PMC12404375; doi:10.1371/journal.pone.0331303)
Supplement: Attachment — Submitted filename: Response to Reviewers.docx [file pone.0331303.s007.docx]

Response to Reviewers for Manuscript PONE-D-25-23758

Title: Mechanism of Huanglian Wendan Decoction in Ameliorating Non-Alcoholic Fatty Liver Disease via Modulating Gut Microbiota-Mediated Metabolic Reprogramming and Activating the LKB1/AMPK Pathway

Reviewer Comments and Author Responses

Response to Comment 1: The source of the plants used in the study, their authentication, and which parts of each plant were used to produce the decoction.

We thank the reviewer for this important suggestion. Detailed information on plant sources, specific plant parts, and authentication protocols has been added to the Preparation of HLWDD subsection. For each herbal ingredient (e.g., Coptis chinensis rhizome from Sichuan, China; Batch CC202305), we now explicitly state: (i) geographical origin, (ii) authenticated plant part, and (iii) batch identification. All specimens were morphologically authenticated by Prof. Tasi Liu (School of Pharmacy, Hunan University of Chinese Medicine) with taxonomic verification via Medicinal Plant Names Service (MPNS; http://mpns.kew.org), and voucher specimens deposited in the University Herbarium (Accession No. HLWD2023-001 to 008). These revisions fully address the request for traceability and comply with ethnopharmacological research standards.

Response to Comment 2: What is the rationale for the concentrations of the medicinal compound used in the experiments? We would expect an acute toxicity test to have been performed.

We sincerely appreciate the reviewer's methodological inquiry. The HLWDD doses (3.6, 7.2, 14.4 g/kg) were rigorously determined through clinical-to-preclinical translation: 1) The high dose (14.4 g/kg) was calculated from the established human clinical dosage of 2.271 g·kg⁻¹·d⁻¹ [Yue et al., 2025] using the FDA-endorsed human-to-rat conversion factor of 6.25 based on body surface area normalization [Reagan-Shaw et al., 2008]; 2) Medium and low doses represented proportional reductions (50% and 25%) to evaluate dose-response relationships, aligning with OECD Guideline 423 for establishing therapeutic ranges prior to chronic toxicity testing. Metformin dosage (90 mg/kg) was directly adopted from published NAFLD intervention studies demonstrating efficacy and safety [Alhamhoom et al., 2023].

Response to Comment 3: Discuss the decoction impact on liver enzymes and skin and insulin sensitivity in discussion section why this decoction is better than available current product and therapeutics

We thank the reviewer for the valuable suggestion. In response, we have revised the Discussion section to clarify the impact of HLWDD on liver enzymes and insulin sensitivity, and to explain its advantages over current NAFLD therapies. Specifically, we added that “Given that AMPK activation is clinically associated with attenuated hepatocellular injury (e.g., reduced ALT/AST) and enhanced insulin sensitivity [25], our molecular findings imply HLWDD may confer similar hepatoprotective and metabolic benefits via AMPK activation.” Furthermore, we emphasized HLWDD’s multi-targeted therapeutic superiority by noting that “HLWDD concurrently resolves inflammation through gut-liver axis modulation while avoiding safety concerns associated with conventional agents such as metformin, pioglitazone, and vitamin E.” These revisions highlight HLWDD’s broader mechanistic action, potential to improve systemic metabolic parameters, and favorable safety profile, thereby addressing the reviewer’s concerns.
